# Supplementary material for: Increasing Notch signaling antagonizes PRC2-mediated silencing to promote reprograming of germ cells into neurons
Source: eLife. 2016 Sep 7;5:e15477. doi: 10.7554/eLife.15477 (PMC5045294; doi:10.7554/eLife.15477)
Supplement: Supplementary file 3. — (A) Information on C. elegans strains used in the study. (B) Information on RNAi clones used in this study. (C) Information on primer design and sequences. DOI: http://dx.doi.org/10.7554/eLife.15477.025 [file elife-15477-supp3.doc]

**Supplementary file 3A:** ***C. elegans* lines used in this study.**

Lab list number or CGC strain names with genotypes.

| **Strain name** | **Genotype / Transgene** | **Reference** |
| --- | --- | --- |
| **BAT28** | *otIs305 (hsp::che-1::3xHA) V; ntIs1 (gcy-5::GFP) V* | Tursun, 2012 |
| **BAT32** | *glp-1(ar202) III.; ntIs1 otIs305 V.* | Tursun,2012 |
| **BAT316** | *glp-1(q175)/hT2 III; otIs305 [hsp-16.2p::che-1::3xHA, rol-6(su1006)] ntIs1 [gcy-5p::GFP, lin-15(+)] V* | this study |
| **BAT317** | *gld-2(q497) gld-1(q485)/hT2 I; otIs305 [hsp-16.2p::che-1::3xHA, rol-6(su1006)] ntIs1 [gcy-5p::GFP, lin-15(+)] V* | this study |
| **#318** | *gld-2(q492) gld-1(q485)(I); glp-1(e2144)* */ hT2(qIs48) (III)* – „Notch OFF” | this study |
| **#6** | *gld-2(q492) gld-1(q485)(I); glp-1(q175)* */ hT2(qIs48) (III)* – „Notch OFF” (used for control strain in the RT-PCR) | this study |
| **#90** | *gld-2(q492) gld-1(q485)(I); glp-1(ar202) / hT2(qIs48) (III)* – „Notch ON” | this study |
| **BS3879** | *gld-2(q497) gld-1(q485)/ hT2::gfp [qIs48] (I); glp-1(q175)/ hT2::gfp [qIs48] (III)* | gift from Tim Schedl |
| **GC833** | *glp-1(ar202) III.* | CGC |
| **GS136** | *glp-1(2144) III.; him-5(e1467)* | CGC |
| **JJ760** | *glp-1(e2144) (III)* | CGC |
| **SS186** | *mes-2(bn11) unc-4(e120) / mnC1 dpy-10(e128) unc-52(e444) (II)* | CGC |
| **#860** | *unc-4(e120) / mIn1(dpy-10(e128)) (II)* | this study |
| **SS222** | *mes-3(bn21) (I)* | CGC |
| **SS360** | *mes-6(bn66) dpy-20(e1282) / nT1(qIs51) (IV)* | CGC |
| **BAT775** | *mes-2(ax2059[mes-2::GFP]) II (CRISPR-Cas9), glp-1(ar202) III.* | this study |
| **JH3203** | *mes-2(ax2059[mes-2::GFP]) II (CRISPR-Cas9)* | CGC |
| **OP591** | *wgIs591 [lag-1::TY1::EGFP::3xFLAG + unc-119(+)]* | CGC |
| **BAT890** | *wgIs591 [lag-1::TY1::EGFP::3xFLAG + unc-119(+)];glp-1(ar202) III.* | this study |
| **CB1282** | *dpy-20(e1282) (IV).* | CGC |
| **BS3538** | *rrf-1(pk1417) I; glp-1(ar202) III.* | gift from Dave Hansen |
| **#786** | *rrrSi185(putx-1::gfp-H”B::tub) II.* | this study |
| **#1258** | *rrrSi181(putx-1::gfp-H”B::tub) II.* | this study |
| **BAT1214** | *glp-1(ar202); rrf-1(pk1417); otIs305 ntIs1* | this study |
| **#793** | *rrSi189(putx-1::FLAG-GFP-linker-TEV::utx-1 ORF+3’UTR)* | this study |

**Supplementary file 3B: Existing RNAi clones used.**

| **Gene name** | **Derived from** |
| --- | --- |
| **empty vector** | addgene |
| ***lin-53*** | Tursun, 2012 |
| ***lag-1*** | Ahringer library |
| ***mes-2*** | Vidal library |
| ***mes-3*** | Ahringer library |
| ***mes-6*** | Ahringer library |
| ***jmjd-1.2*** | Ahringer library |
| ***jmjd-2*** | Ahringer library |
| ***jmjd-3.1*** | Ahringer library |
| ***jmjd-3.2*** | Ahringer library |
| ***jmjd-3.3*** | Ahringer library |
| ***C07G1.6*** | Ahringer library |
| ***C07G1.7*** | Ahringer library |
| ***aldo-1*** | Ahringer library |
| ***tag-123*** | Ahringer library |
| ***lin-15B*** | Ahringer library |
| ***B0416.6*** | Ahringer library |
| ***F20D1.1*** | Ahringer library |
| ***ZC84.3*** | Ahringer library |
| ***T27F6.4*** | Ahringer library |
| ***mek-1*** | Ahringer library |
| ***utx-1*** | Ahringer library |

**Supplementary file 3C:** **List of Primers**

Primers used to generate RNAi clones were equipped with HindIII recognition site for cloning into the feeding vector.

utx-1RNAi tgtAAGCTTaatcggaatcgttcaagcac

utx-1RNAi tgtAAGCTTacacttcacactcgcacgtc

ZC84.3LEFT tgtAAGCTTggaaggaatcaagaaactgttg

ZC84.3RIGHT tgtAAGCTTttccattttcgagtccatttc

spat-3LLEFT tgtAAGCTTagaaatgcggtcatcgattc

spat-3LRIGHT tgtAAGCTTttccggatgtccatctcttc

spat-3BLEFT tgtAAGCTTgcagttcctcagaagccaac

spat-3BRIGHT tgtAAGCTTcaatcttgccgcttccttag

nhr-48LEFT tgtAAGCTTccgaatacgtcaacacaacg

nhr-48RIGHT tgtAAGCTTgtgcacacatcccacagttc

lin-15BLEFT tgtAAGCTTacctgagccagagagaaacg

lin-15BRIGHT tgtAAGCTTaagtgcacgtcgttgagatg

lin-15ALEFT tgtAAGCTTtctgcagctcacatgtttcc

lin-15ARIGHT tgtAAGCTTagacccattgaccatccttg

gpd LEFT tgtAAGCTTagccaagtgtcggaatcaac

gpd RIGHT tgtAAGCTTgtggcgatgtacgagatgag

tag-123LEFT tgtAAGCTTcgaggaagatgctcaagagg

tag-123RIGHT tgtAAGCTTatccgatcgttccagtcatc

lsy-2LEFT tgtAAGCTTtcaaatggggtttcctgaag

lsy-2RIGHT tgtAAGCTTgctggctgaggaacaatttc

wht-9LEFT tgtAAGCTTgctgtggagaacctgaaagc

wht-9RIGHT tgtAAGCTTcccttctccttaaccgatcc

Primers used for the microarray analysis validation by RT-qPCR:

At least one primer in each pair is specific for an exon-exon junction.

C07G1.7P LEFT GAGGTGTTCCTTTCGATAGAG

C07G1.7P RIGHT TCTCGTTCATCTGATGAGTGG

T27F6.4P LEFT ATCAAGGCAAAGGAATCAAGC

T27F6.4P RIGHT CTTCTTCAACTCCAACAGGG

F20D1.1P LEFT TCTTCTTCAAATGACAGCTGG

F20D1.1P RIGHT GCACAGGAAGATTGGAATGAG

C55C3.6P LEFT TGATGGAAAGGACAAGAAGG

C55C3.6P RIGHT TTGTCTCGAATAGAACACAACC

dpy-23P LEFT TGATCCAAACAAAGCAAGCC

dpy-23P RIGHT CTTAGTGGTACGGTATCTCATCAG

R08R3.3P LEFT CTCTAGACCAGGGTCATCAG

R08R3.3P RIGHT GGTTTCCGGAGTATTATTTGGC

C15H9.9P LEFT GGTGTTAGTGCCAATATCTC

C15H9.9P RIGHT AAAGAACTCCTTCTACGCTC

T25D12.5P LEFT CATGCTAACTGGTTCTTCGT

T25D12.5P RIGHT GGTGTCTGAACATCGTTGTC

cki-2P2 LEFT ATGAGTCGTTCAGTTTCAATCG

cki-2P2 RIGHT GATGCAACAAATCTAAGTGGC

utx-1e fw CGAGCCTAGTGTTAGTGGAC

utx-1e bw GCACATATGAATGCCTGAAGAG

tbb-2 f 495 for tubulin ( Kalchhause*r et a*l. 2011)

tbb-2 r 696 for tubulin (Kalchhause*r et a*l. 2011)

Primers used for the CHIP-qPCR:

Primer for qPCRs were designed using Primer3Plus (Untergasse*r et a*l. 2007) with the following settings: max. amplified region 200 bp, min. 100 bp; GC content: 50-60%; min. primer length: 18 nt, max. length 24 nt; melting temperature: min. 58°C, max. 63°C; max. 3' self complementary allowance set to 1; max. allowed length of a mononucleotide repeat (max. poly-x): 3.

aldo-1 LEFT     GCTCCTCTCGCTTCTTCAAA

aldo-1 RIGHT     AAGAGCCGGAGCTTTCTCTC

B0416.5 LEFT CCTGGAAACAAAGAGACCCAGT

B0416.5 RIGHT AAGAGCGTCCTGGCATCTAA

C07G1.7 LEFT CTTCCAGGCGTGTACACAAA

C07G1.7 RIGHT TGCCTATTTACTCGCGCTTC

ceh-20 LEFT CCAGATCTTCCGAACACCTACT

ceh-20 RIGHT TGAGATTGGCTGGGTGTGT

F20D1.1 LEFT CCGCTTCTGTTTCACTTCCT

F20D1.1 RIGHT CCCATGTCGACAGAGAACAA

lst-1 LEFT GACAACTTTCCCACGCTTGT

lst-1 RIGHT CTCGCGCGAAGATTGAATAG

lst-1 (3’UTR) LEFT     AATTGGGATGAGGCACACAG

lst-1 (3’UTR) RIGHT    AGAGTTCGCGAGATGTGGAT

lst-1 (3’UTR) LEFT GTCGAACAAATGGGACACG

lst-1 (3’UTR) RIGHT GTGTGTGTGGCAGGAGCTAA

nkat-3 LEFT GGTATATCCCGGCAATTGTG

nkat-3 RIGHT TGAGAGACAGGCTGGAAGAGA

sygl-1 LEFT GCAGTGTGTTCTGCCGAAAT

sygl-1 RIGHT AATCCACTGAAGACGCCACT

utx-1 LEFT CAATCCATACTTGCGCACAC

utx-1 RIGHT CCCGTCTCTACTGTGCCTGT

Primer pairs used to generate probes from cDNA for *in-situ* hybridization for *utx-1.*

utx-1 is New L cggtgactgtgaatggtttg

utx-1 is New R acacttcacactcgcacgtc

utx-1 is New L S TAATACGACTCACTATAGGGACTcggtgactgtgaatggtttg

utx-1 is New R AS TAATACGACTCACTATAGGGACTacacttcacactcgcacgtc

**REFERENCES**

Fox PM, Vought VE, Hanazawa M, Lee M-H, Maine EM, Schedl T (2011) Cyclin E and CDK-2 regulate proliferative cell fate and cell cycle progression in the C. elegans germline. *Development (Cambridge, England)* **138**, 2223–2234. doi:10.1242/dev.059535.

Gartner A, MacQueen AJ, Villeneuve AM (2004) Methods for analyzing checkpoint responses in Caenorhabditis elegans. *Methods in molecular biology (Clifton, NJ)* **280**, 257–274. doi:10.1385/1-59259-788-2:257.

Kalchhauser I, Farley BM, Pauli S, Ryder SP, Ciosk R (2011) FBF represses the Cip/Kip cell-cycle inhibitor CKI-2 to promote self-renewal of germline stem cells in C. elegans. *The EMBO Journal* **30**, 3823–3829. doi:10.1038/emboj.2011.263.

Patel T, Tursun B, Rahe DP, Hobert O (2012) Removal of Polycomb Repressive Complex 2 Makes C. elegans Germ Cells Susceptible to Direct Conversion into Specific Somatic Cell Types. *Cell Reports*. doi:10.1016/j.celrep.2012.09.020.

Tursun B, Patel T, Kratsios P, Hobert O (2011) Direct conversion of C. elegans germ cells into specific neuron types. *Science (New York, NY)* **331**, 304–308. doi:10.1126/science.1199082.

Untergasser A, Nijveen H, Rao X, Bisseling T, Geurts R, Leunissen JAM (2007) Primer3Plus, an enhanced web interface to Primer3. *Nucleic Acids Research* **35**, W71–4. doi:10.1093/nar/gkm306.
